# Tirzepatide for weight and behavior management in a patient with Smith-Magenis syndrome

**DOI:** 10.1210/jcemcr/luag152

**Published:** 2026-06-03

**Authors:** X Charlene Liao, Tao Huang, David S Hong, Liqun Luo

**Affiliations:** Independent Scholar, Palo Alto, CA 94303, USA; Independent Scholar, Mountain View, CA 94041, USA; Department of Psychiatry and Behavioral Sciences, Stanford University, Stanford, CA 94305, USA; Howard Hughes Medical Institute, Department of Biology, Stanford University, Stanford, CA 94305, USA

**Keywords:** Smith-Magenis syndrome, tirzepatide, syndromic obesity, behavior management, therapeutic strategy

## Abstract

Smith–Magenis syndrome (SMS) is a rare neurodevelopmental disorder characterized by intellectual disability, behavioral dysregulation, and hyperphagia-driven obesity. While dual glucose-dependent insulinotropic polypeptide (GIP) and glucagon-like peptide-1 (GLP-1) receptor agonists have demonstrated efficacy in the general population, their use in patients with SMS has not been described. We report the case of a 31-year-old woman with SMS (17p11.2 deletion) treated with tirzepatide, a dual GIP/GLP-1 receptor agonist. The patient presented with lifelong obesity (body mass index [BMI] 32.0-32.9 kg/m^2^ in adulthood) and aggressive behaviors refractory to standard management. Following initiation of tirzepatide, titrated to 5 mg weekly, she achieved 9.4% weight loss (7.3 kg) over 10 months, along with improvement in fasting glucose levels. Concurrently, caregivers reported notable behavioral improvements, including reduced food-seeking behavior and impulsivity. Quantitative analysis demonstrated a significant reduction in aggression. The treatment was well tolerated. This case suggests that tirzepatide may represent a promising therapeutic option for SMS, targeting both metabolic and central nervous system pathways involved in its phenotype.

## Introduction

Smith-Magenis syndrome (SMS) is a complex neurodevelopmental disorder that presents significant clinical challenges [1, 2]. The syndrome is caused by a heterozygous interstitial deletion of approximately 3.7 Mb on chromosome 17p11.2 [2], encompassing the retinoic acid-induced 1 (*RAI1*) gene. Pathogenic variants of *RAI1* are responsible for about 10% of SMS cases [3]. Haploinsufficiency of *RAI1* drives the core features of the syndrome, including maladaptive behaviors such as self-injury, aggression, and impulsivity [4-6].

A cardinal feature of SMS is early-onset obesity coupled with profound food-related behavioral problems, the severity of which rivals that observed in Prader-Willi syndrome (PWS) [7]. Consequently, traditional weight management strategies, including lifestyle and behavioral interventions, often have limited success.

In recent years, entero-pancreatic hormone-based treatments have revolutionized obesity management [8]. Tirzepatide represents an evolution in this class, as a single molecule acting as a dual agonist for both glucose-dependent insulinotropic polypeptide (GIP) and glucagon-like peptide 1 (GLP-1) receptors [9, 10]. This dual mechanism provides synergistic effects on glucose and weight regulation, leading to superior outcomes compared to selective GLP-1 receptor agonists in large-scale trials [11-13]. Here, we present a case report on the use of tirzepatide for the management of weight and behavior in an adult with SMS.

## Case presentation

The patient was a 31-year-old female from the United States with a genetically confirmed diagnosis of SMS due to the 3.7 Mb common deletion in 17p11.2 [2]. The patient lived at home with family members at the time of this case report and attended an adult day program from Monday to Friday (8:30 Am–4:30 Pm).

The patient's medical history was notable for lifelong, progressive obesity beginning in early childhood and resistant to structured dietary plans, physical activity, and behavioral therapy. Her weight continued to increase over time, leading to complaints about foot pain, refusal to exercise, such as walking on a treadmill, and metabolic complications, including dyslipidemia and impaired fasting glucose. Notably, the patient was not on cholesterol-lowering medications, and medical history was negative for heart failure, epilepsy, microvascular disease, and bone anomalies (other than brachydactyly). The patient's symptoms also included sleep disturbances, hearing loss, and myopia, all typical of SMS. She took aripiprazole (2.5 mg in the morning and 5 mg at night) to control aggressive behaviors and ramelteon (8 mg at night) to manage her sleep. These medications had been used for years without impact on her weight and were continued without changes throughout the observation period.

## Diagnostic assessment

The patient's height was 155.6 cm. Prior to the initiation of tirzepatide treatment, the patient's weight was 77.6 kg, with a BMI of 32.0 kg/m^2^. Routine monitoring laboratory tests for vitamin D, thyroid hormone, and kidney and liver function were within normal limits. Throughout her life, the patient had consistently demonstrated slightly higher than normal total cholesterol levels. Fasting blood glucose level was measured at 14 months and 6 months prior to tirzepatide treatment and showed slightly elevated levels of 103 mg/dL (5.7 mmol/L) and 99 mg/dL (5.5 mmol/L) (normal range, 70-99 mg/dL [3.9-5.5 mmol/L]), respectively.

At baseline, the patient was verbal and communicative, with developmental asynchrony and a specific pattern of cognitive strengths and weaknesses typical of SMS. The patient's behavioral phenotype was also consistent with SMS [7], characterized by rigid, inflexible demands placed on others and external circumstances, obsessive behaviors, severe impulsivity, low frustration tolerance, aggressive outbursts, and self-injurious behaviors such as head-hitting. A particularly challenging feature was profound hyperphagia; the patient exhibited constant food-seeking behaviors and extreme frustration when denied access to excess or unhealthy food. These behaviors necessitated close supervision and a home-based token economy system to manage aggression and food demands.

## Treatment

Given the severity of weight symptoms and the failure of prior interventions, tirzepatide was initiated with the rationale of utilizing its dual-agonist mechanism for weight loss [14] and its central effects on appetite signaling to ameliorate hyperphagia [8]. Treatment began on January 10, 2025 (Week 1), with a subcutaneous injection of 2.5 mg tirzepatide weekly for 12 weeks, escalating to a maintenance dose of 5 mg tirzepatide weekly thereafter. As of the data cutoff date at Week 42, the patient had been treated with 5 mg tirzepatide weekly for 30 weeks. For steady-state comparison, data analysis excludes the 12-week titration period on 2.5 mg tirzepatide, except for weight monitoring. There had been no prior pharmacological treatment for weight management, such as phentermine-topiramate or other medications. Lifestyle guidance had been consistently provided throughout the patient's life as part of routine behavioral management and was not specific to this observation period.

## Outcome and follow-up

**Figure 1:**
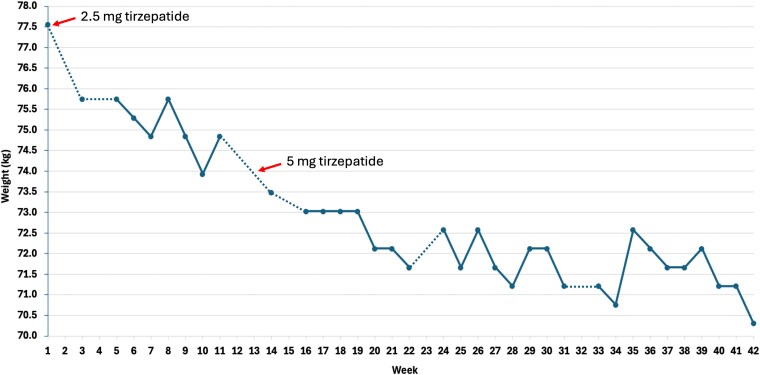
Longitudinal changes in body weight based on weekly measurements. The patient's body weight was measured weekly, beginning on January 10, 2025 (Week 1), prior to initiation of tirzepatide at 2.5 mg. On April 4, 2025 (Week 13), the tirzepatide dose was increased to 5 mg weekly, continuing through the behavioral data cutoff date of October 31, 2025 (Week 42). Red arrows indicate the timing of initiation of weekly tirzepatide injections at 2.5 and 5 mg. Each dot represents a weight measurement for a given week. Dotted lines indicate missing data points (Weeks 2, 4, 12, 13, 15, 23, and 32) between otherwise weekly measurements.

Over the 10-month observation period, the patient demonstrated consistent and steady weight reduction. Her weight decreased from a baseline of 77.6 kg (BMI 32.0 kg/m^2^) to 70.3 kg (BMI 29.0 kg/m^2^), representing a total loss of 7.3 kg or 9.4% of her initial body weight (Fig. 1). These data were based on weekly weight measurements using the same scale at the same time of the week (prior to weekly tirzepatide injection). Concurrently, her fasting glucose levels improved from 103 and 99 mg/dL (5.7 and 5.5 mmol/L), measured 14 and 6 months prior to initiation of treatment, respectively, to 89 mg/dL (4.9 mmol/L), measured 3 months after initiation of treatment. There was no decrease in her fasting cholesterol level.

Caregivers reported notable behavioral improvements, including a substantial reduction in both the intensity and frequency of food-seeking behaviors, as well as the emergence of post-meal satiety. The patient was described as “calmer” and “more content,” contributing to improved social interactions and enhanced family quality of life.

**Figure 2:**
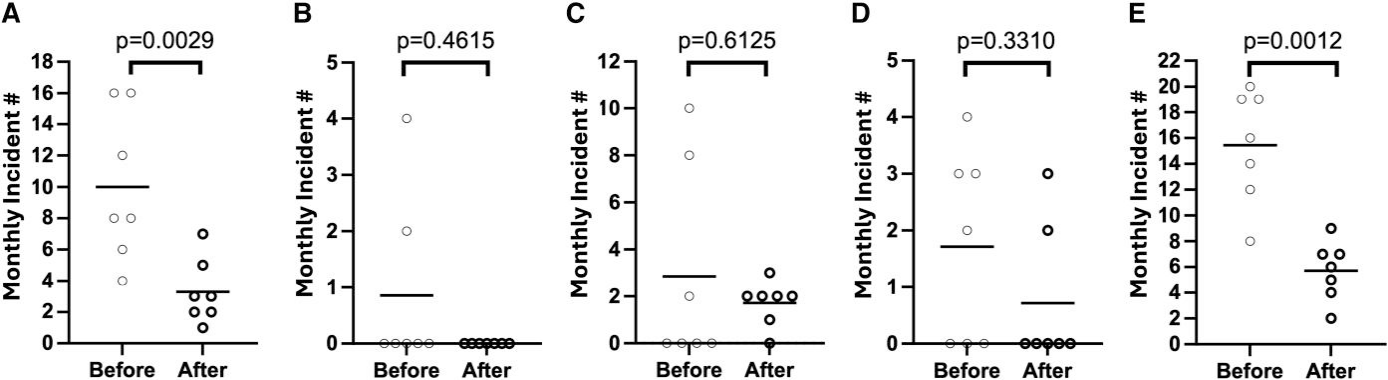
Changes in maladaptive behaviors at the adult day program. Monthly incidents (Y-axis) were used to track behaviors including hitting others (A), pulling hair (B), slapping others (C), self-injurious behavior (D), and total aggression (E; encompassing all behaviors in A–D). “Before” includes the 7 months immediately preceding initiation of tirzepatide treatment (June–December 2024). “After” includes the 7 months following maintenance treatment with tirzepatide at 5 mg weekly (April–October 2025). Data are presented as monthly incident counts, with each month represented as a data point in the plots. Horizontal bars indicate the mean. The Mann–Whitney U test was used for statistical comparisons.

Quantitative analysis of monthly behavior charts completed by staff at the patient's adult day program supported these qualitative observations. Her maladaptive behaviors were described using phrases such as “hitting others” (intentional physical aggression directed toward specific individuals), “pulling hair” (also directed toward others), “slapping others” (largely undirected, rapid movements of her arms and hands that impacted nearby individuals), and “self-injurious behaviors” (including hitting her head with her hands or against a wall or floor, as well as biting her hands or wrists). The frequency of “hitting others” was significantly reduced during the 7 months following maintenance treatment with 5 mg weekly tirzepatide (“after”), compared with the 7 months immediately preceding initiation of tirzepatide treatment (“before”; see Fig. 2A). The frequencies of 3 other aggressive behaviors—“pulling hair,” “slapping others,” and “self-injurious behaviors”—also trended lower after treatment, but these differences were not statistically significant compared with the pre-treatment period (Fig. 2B–2D). The incidence of total aggression (including all 4 categories above) was also significantly reduced after tirzepatide treatment (Fig. 2E).

The patient tolerated tirzepatide well. She did not experience discomfort or common adverse side effects associated with this class of medications throughout the 10-month treatment period.

## Discussion

This case study describes the use of the dual GIP/GLP-1 receptor agonist tirzepatide in an individual with SMS. The primary findings are 2-fold: the medication induced sustained weight loss (9.4% over 10 months) and was associated with significant improvement in the patient's neurobehavioral phenotype. The observed weight reduction aligns with outcomes reported in larger clinical trials of tirzepatide for obesity using a 5 mg dose over 6-12 months [11, 12, 14]. These include (1) an 11% reduction in body weight among patients with type 2 diabetes (T2D) [11]; (2) a 10% reduction after 6 months and 15% after 12 months in a retrospective analysis of US adults with obesity [12]; and (3) an estimated 14% reduction over 42 weeks with a maintenance dose of 5 mg in a dedicated prospective study of once-weekly tirzepatide for the treatment of obesity [14]. In this patient, fasting glucose decreased from slightly elevated levels (103 mg/dL [5.7 mmol/L] and 99 mg/dL [5.5 mmol/L]) prior to treatment to a normal level (89 mg/dL [4.9 mmol/L]) after treatment. These weight and metabolic improvements are clinically significant and may reduce future risk of type 2 diabetes and cardiovascular disease [15, 16].

Achieving 9.4% weight loss over a relatively short period on a low dose of tirzepatide (12 weeks at 2.5 mg followed by 30 weeks at 5 mg) in a patient with SMS is particularly noteworthy, given reported failures of other targeted obesity therapies, such as the melanocortin-4 receptor (MC4R) agonist setmelanotide, to produce significant weight loss in this population [17]. This observation suggests the GIP/GLP-1 pathway may be a more effective target, and individuals with SMS could retain response to GIP/GLP-1 agonists. A recent case report of the GLP-1 receptor agonist semaglutide in a young adult with SMS also described improvements in weight and impulsivity [18]. Together with the present case, this supports a potential targeted effect of incretin-based therapies for individuals with SMS.

The obesity phenotype in SMS [2, 7] is rooted in the haploinsufficiency of *RAI1* [3]. Preclinical studies show that mouse models with heterozygous *Rai1* knockout develop hyperphagia, impaired satiety, and early-onset obesity [19-23]. Neurogenetic analyses in mouse models further highlight the role of hypothalamic neurons in obesity [4]. A recent mouse study demonstrated that GLP-1–expressing neurons in the hindbrain act in the hypothalamus to regulate appetite [24]. Our data suggest that tirzepatide might overcome dysfunctional regulatory control of appetite in hypothalamic neurons due to *RAI1* haploinsufficiency.

Perhaps more impactful than the weight loss were the behavioral changes. Reductions in food-seeking behavior, impulsivity, and aggression addressed the most debilitating aspects of the SMS neurobehavioral phenotype [7, 25]. We hypothesize that by targeting central pathways regulating appetite and satiety, tirzepatide reduced the patient's persistent preoccupation with food. The reduced perseveration around food likely removed a major trigger for frustration and subsequent aggressive outbursts.

These findings may have implications for other neurodevelopmental disorders associated with syndromic obesity, such as PWS [26], which shares a similar hyperphagic and behavioral phenotype with SMS [7]. Our findings suggest that GIP/GLP-1 agonism could be a therapeutic strategy for PWS, a possibility currently under investigation in a clinical trial (NCT06901245).

This case provides promising evidence that GIP/GLP-1 agonism may be a valuable therapeutic strategy for SMS, warranting further study through prospective case series or clinical trials to systematically evaluate efficacy and safety in this population.

## Learning points

- This case study provides evidence that tirzepatide may be an effective treatment for both obesity and certain challenging behavioral symptoms associated with Smith-Magenis syndrome.
- By targeting the intense food preoccupation that drives much of the challenging behavior, tirzepatide appears to offer a dual benefit that could improve the quality of life for individuals with SMS and their families.
- This study, which reports more pronounced weight loss over a shorter period as well as quantitative behavioral profiling, substantiates findings from a recent case report on the GLP-1 receptor agonist semaglutide in a patient with SMS [18], particularly regarding weight loss and behavioral impulsivity.
- If these preliminary findings are confirmed by a case-control or cohort study, tirzepatide could represent a therapeutic strategy in the management of SMS and potentially other neurodevelopmental disorders characterized by syndromic obesity and intractable behavioral challenges.

## Data Availability

Original data generated and analyzed during this study are included in this published article.
